# Light People: Professor Cheng-Wei Qiu

**DOI:** 10.1038/s41377-023-01138-x

**Published:** 2023-04-10

**Authors:** Tingting Sun

**Affiliations:** Light Publishing Group, Changchun Institute of Optics, Fine Mechanics and Physics, Chinese Academy of Sciences, 3888 Dong Nan Hu Road, Changchun, 130033 China

**Keywords:** Metamaterials, Photonic devices

## Abstract

“Mind-controlled information transmission” is one of human’s ultimate fantasies for the development of science and technology. On June 11, 2022, *eLight* Journal simultaneously published two research papers on brainwave-based metasurfaces, and proposed a new concept of metasurface controlled by mind for the first time in the world. This major scientific breakthrough rushed up to the hot search list of public media. It was read more than ten million times, and the related video has been played 600,000 times, which aroused the audience’s strong interest and discussion, and really made a significant leap toward “mind control”. We are greatly honored to invite Prof. Cheng-Wei Qiu from National University of Singapore, the corresponding author of both above-mentioned articles and the co-Editor-in-Chief of *eLight* Journal, to have an exclusive interview. Prof. Cheng-Wei Qiu showed us the profound interpretation of his research works and shared his unique insights on journal development and talent cultivation.

## Abstract (web summary)

Prof. Cheng-Wei Qiu spoke about the structured surface of multi-dimensional fusion and beam manipulation.

## Graphical abstract

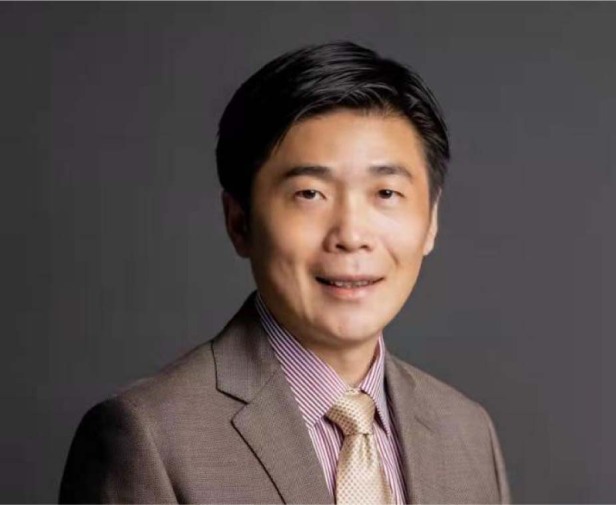

**Figure:**
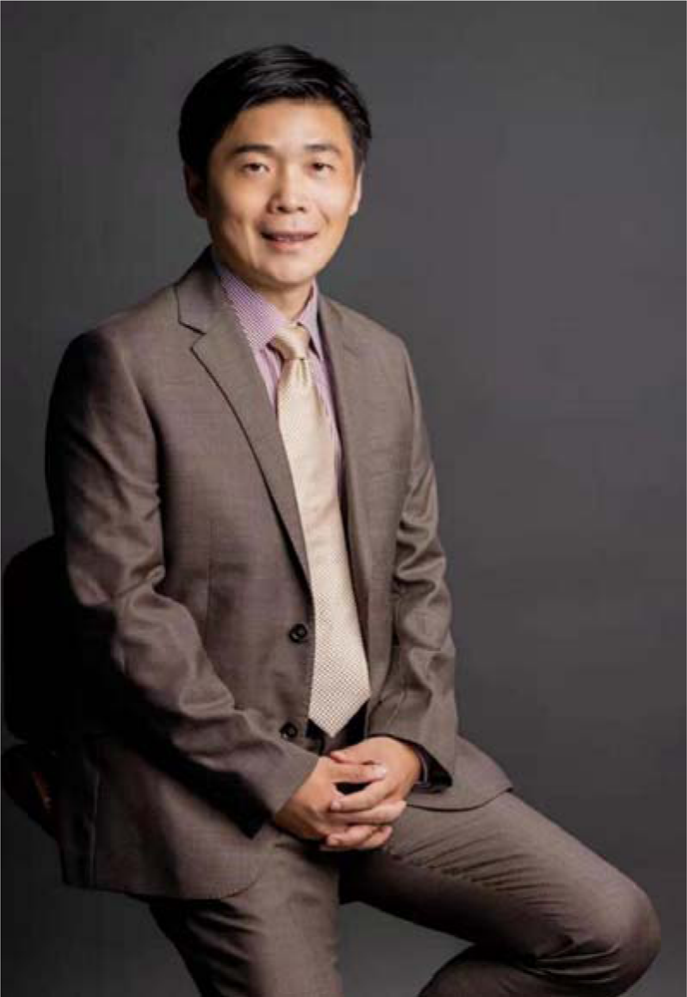

**Biography:** Professor Cheng-Wei Qiu is the Dean’s Chair Professor of NUS, and a fellow of Optica (formerly OSA), SPIE, and The Electromagnetics Academy, US. He received his B.Eng. degree from USTC in 2003 and Ph. D. degree from NUS in 2007, respectively. He was a Postdoctoral Fellow at Physics Department in MIT thereafter. He joined NUS as an Assistant Professor in December 2009, and was promoted to Associate Professor in January 2017. Since January 2018, he was promoted to Dean’s Chair Professor at Faculty of Engineering, NUS. He was the recipient of SUMMA Graduate Fellowship in Advanced Electromagnetics in 2005, IEEE AP-S Graduate Research Award in 2006, URSI Young Scientist Award in 2008, NUS Young Investigator Award in 2011, MIT TR35@Singapore Award in 2012, Young Scientist Award by Singapore National Academy of Science in 2013, Faculty Young Research Award in NUS 2013, SPIE Rising Researcher Award 2018, Young Engineering Research Award 2018, and Engineering Researcher Award 2021 in NUS, World Scientific Medal 2021 by Institute of Physics Singapore, and Achievement in Asia Award (Robert T. Poe Prize) 2023 by International Organization of Chinese Physicists and Astronomers. His work has been selected as Top 10 Breakthroughs 2020 by Physics World, and Optics in 2021 by Optica. As an overseas partner, he won the China’s Top 10 Optical Breakthroughs for 4 times. His research is well known for the structured surface of multi-dimensional fusion and beam manipulation. He has published over 460 peer-reviewed journal papers in *Nature*, *Science*, *Light: Science & Applications (Light)*, *eLight*, and other top journals. He has been selected as Highly Cited Researcher by Web of Science for 4 consecutive years from 2019 to 2022. At present, Prof. Qiu serves as co-Editor-in-Chief of *eLight*. He has also served as an editorial board member and advisors for several journals including *Light: Science and Applications*, *Laser and Photonics Review, Advanced Optical Materials, ACS Photonics, PhotoniX*, and *Photonics Research*.

**Q1: Can you briefly introduce the main research ideas of the work “mind-controlled metasurface** ^1,2^ **” on Weibo hot search list? What positive impact will this major development bring to our real life?**

**A1:** Our original intent in developing mind-controlled metasurface is to open up an unprecedented path. Generally, the main methods to actively control metasurface include electronic control, optical control, and mechanical control, etc. However, we wanted to attempt one of the most challenging solutions: Can we use brainwaves to control the metasurface? That was our initial idea, after nearly 7 years, we eventually demonstrated the prototype. The P300 brainwave signals will have different responses to different stimulus, so the changes of the environment and stimulus captured by naked eyes will be reflected in the brainwaves. We thereby exploit brainwaves to control the output voltage of every pixel on the metasurface, and the whole process is completely wireless and controlled by people’s mind. This development has some meaningful practical applications, for example, for some people with physical disabilities, they can control simple auxiliary medical devices by their thoughts when they cannot move easily. Especially for terminal medicine or precision medicine, you can transmit your information through your own thinking. It is particularly valuable in the microwave field of mind-controlled metasurface, because microwave can be transmitted at long distances without any wiring connections, which is also very neat. We still work dedicatedly in this direction, for example, we have done some experiments to verify the feasibility of employing eye motion to control UAV to establish directional information channel. This method can control the machines through the eyes, and the machines can in turn see other scenes beyond the line-of-sight of human eyes. Therefore, “mind-controlled metasurface” not only exhibits great advancement in creativity, but also showcases important value in practical applications, including communication, remote customer service, medical care, and some personalized precision medicine in the future.

**Q2: As a kind of polarized light, circularly polarized light (CPL) has been widely used in many fields such as imaging, quantum optics, and drug analysis. Currently, what are the main difficulties in detecting CPL? Your team proposed a new design concept for optoelectronic detection devices from the perspective of geometry and symmetry**^3^, **which was published in** ***Nature Photonics*. What is the major breakthrough in this work? In the future, how can we realize the integrated on-chip detection of multi-dimensional information such as polarization, wavelength, and angular momentum? How do you think of the prospects of this direction?**

**A2:** Be it linear or circular polarization, the traditional detection methods need a large area to place various wave plates and detectors, and obtain the polarization information after interference, which leads to a very large volume of the whole system and hence not miniaturized or portable. Previous studies have not paid much attention to the geometric effects. We proposed to realize integrated on-chip and highly sensitive polarization photodetectors by using geometric arrangement of photodetectors. The major breakthrough is that we have shrunk the whole system originally placed on an optical platform at the scale of meter, to a system of millimeters or even hundreds of microns. In addition, instead of measuring light by light, we converted light into electricity and obtain the polarization information from reading the electrical output. Such on-chip optoelectronic devices not only break the limits of traditional polarization detection system in integration, volume, and portability, but also work under room temperature in the infrared regime. Many commercial infrared photodetectors unfortunately rely on the cooling apparatus. From the view of geometry and symmetry, our work proposes a Lego-like playground to enable multiple function arrangements, that is, different designs can be placed in the same area to detect linear polarization, circular polarization, and even spectrum. We are currently still trying to design some orbital angular momentum (OAM) detection along this line.

I think it is a promising path to realize multi-dimensional detection through geometric arrangements of photodectors. There are two main approaches. First, it is the multi-dimensional detection, by blocking function areas in time or space to realize individual function detection. The other approach is multiplexing, with two possibilities. The first one is that it just happens to find a very unique geometric distribution, where the responses to different polarization or OAM are sufficiently distinct. So it can be read and resolved. The second is through machine learning. When doing high-dimensional photodetection, only electrical signals are collected. But we require a large enough database so as to extract various characteristic information carried by incident light from electric read-out, which can be achieved through machine learning or deep learning.

**Q3: Your team made a significant progress in the cross field of micro-nano optics and chiral optics. The chiral bound states in continuum (BIC) with extreme intrinsic chirality was realized and observed for the first time, which was published in** ***Nature*** **with the title “Observation of intrinsic chiral bound states in the continuum**^4^**”. What’s innovative about this work? What are the potential applications?**

**A3:** Chirality is a research field with long-lasting charms. Chiral BIC has been captivated as a *Holy Grail* in the field of chiroptics, because it is totally decoupled from circularly polarized light (CPL) of one handedness while strongly interacts with the other, manifested by unity circular dichroism (CD) and high quality (*Q*) factor. It is so alluring that many works, if not all, have claimed to nail it recently and a simple search will pop up a long list of such works including top-forum publications. The *inconvenient truth* is that none of them actually got close to the summit. Most of the previous BIC-aided attempts took extrinsic pathways, such as oblique incidence and structural anisotropy, to realize strong chiroptical performance. A few theoretical works^5–7^ have been recently presented to directly achieve the intrinsic chiral BIC per se. Those insightful proposals resort to three-dimensional (3D) sophisticated geometries, which are too challenging to be made in optical frequency. This is why the true “intrinsic chiral BIC” is only accessible in theory.

Our work takes a paradigm shift as a theoretical “pavement” to experimentally reach the said *Holy Grail*. We report the first, true and direct recipe to intrinsic chiral BIC by introducing slant-perturbation metasurfaces to break both in-plane and out-of-plane symmetry at one go. A tiny perturbation in slant angle of the metasurface, enabled by our home-made slant-etching nanotechnology, leads to the significant breakthrough in demonstrating intrinsic chiral BIC in visible light. It simultaneously showcases a near-unity CD of 0.93 and a record-high *Q*-factor of 2663 in visible. The possible influence of structural anisotropy has been ruled out, and we confined ourselves in the normal incidence. To further demonstrate the intrinsic chirality of the generated chiral BIC mode, we have conducted chiral emission experiments based on the slant-perturbation metasurface sample. Dye molecules are spin-coated on the slant-perturbation metasurface, and pumped by a 400 nm CW laser. The emission band of the dye molecules spectrally matches with the resonant wavelength of the chiral BIC, and the measured photoluminescence (PL) spectrum exhibits CD as large as 0.9. The emission peak of RCP PL is very sharp due to the high *Q*-factor of chiral BIC, while no emission peak is observed for LCP PL, owing to the intrinsic chirality of chiral BIC. According to the chiral emission results, intrinsic chiral BIC with true chirality is fundamentally distinct from those BIC states with extrinsic chirality.

This is the first piece for the true intrinsic chiral BIC in the visible frequency and with extremely high *Q*. It is a different ball game compared to those extrinsic claims or theoretical proposals. The enabling technology is unique and the physics model is elegant: a perturbation in symmetry solves all. Our concept and recipes can be readily extended to infrared and even longer wavelengths, promising future applications in chiral light sources and detectors, chiral sensing, quantum optics, and asymmetric photocatalysis.

**Figure:**
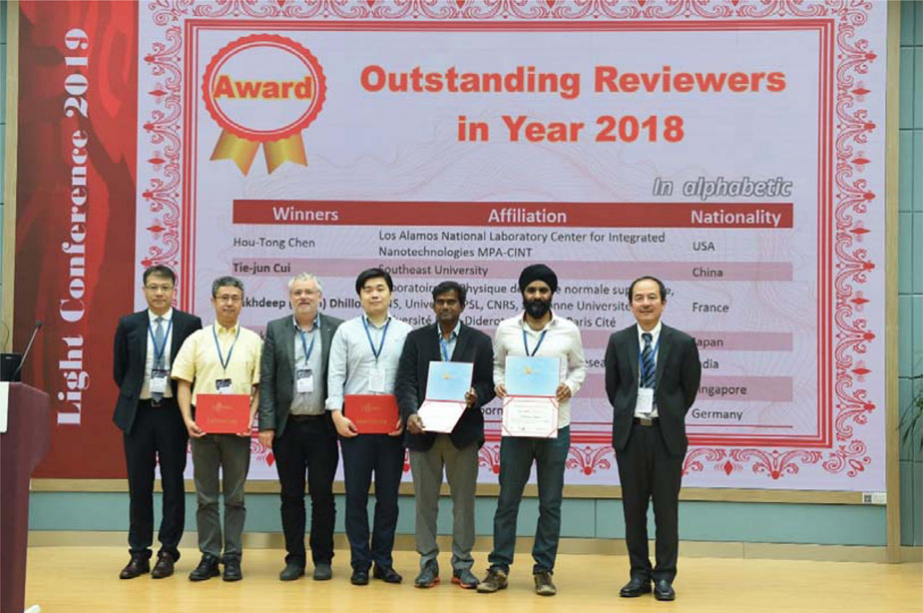
Prof. Cheng-Wei Qiu won 2018 Light Outstanding Reviewer Award

**Q4: As an information carrier, optical quantum has developed rapidly in many fields due to its unique advantages. Miniaturization and integration are the only way for optical quantum technology to realize practical applications. At present, what are the problems in constructing on-chip integrated entangled quantum light sources? Your latest work has proposed an ultra-thin nonlinear quantum source** ^8^ **. What is the key to achieve this result?**

**A4:** The construction of on-chip integrated entangled quantum light source is a scientific research project facing the national major needs. The biggest challenge is the balance of efficiency and volume, which are natively excluding each other. Hence, to make the source thinner means poorer efficiency. For the first time, we used NbOCl_2_ material of only 46 nm thick to realize a quantum light source of spontaneous parametric down-conversion (SPDC). The key of this work is that the interlayer electrons of the van der Waals material are weakly coupled. What is particularly interesting is, from the perspective of monolayer, the second-order nonlinear χ^(2)^ of this material is basically close to monolayer of the transition metal dichalcogenides (TMDCs). However, when no matter how many layers are stacked, the χ^(2)^ is always reserved, that is, the thickness can be used as a tool to enhance the nonlinear light-matter interaction. Compared with the commonly used lithium niobate (LiNbO_3_) or other BBO crystals similar to LiNbO_3_, using NbOCl_2_ as a nonlinear quantum light source combines the advantages of TMDCs and nonlinear LiNbO_3_ crystals. Its χ^(2)^ is corresponding to TMDCs monolayer and higher than LiNbO_3_, and it doesn’t have to be as thick as LiNbO_3_. This material is a gift from nature. We can achieve ultra-thin nonlinear quantum light sources by constructing a relatively thin layer and maintaining a very high χ^(2)^. This is very important for future ultra-thin and light-loaded entangled quantum light sources. We are also doing some follow-up research to incorporate polarization entanglement. Apart from NbOCl_2_, can we do with other materials? The answer is yes. We can also make ultra-thin quantum light sources by nano-patterning other materials to achieve high χ^(2)^. Be it in quantum communication or quantum optics, the source is always pivotal. We have provided a proof-of-concept recipe, hoping to support the whole quantum optics research field in the next few years.

**Q5: Polaritons is not only the cross disciplinary frontier field of condensed matter physics, optical physics, and materials science, but also one of the traditional advantage research directions of China. You proposed and experimentally proved the existence of ghost hyperbolic polaritons electromagnetic waves in traditional birefringent crystal**^9,10^, **breaking the inherent understanding of polariton classification. Could you introduce the characteristics of this ghost polariton? What are the technical difficulties to be solved in the follow-up research of this work?**

**A5:** Polariton is a quasiparticle produced by the strong coupling of light and matter. It has both the properties of light and matter. This is a very interesting field, and also one of the traditional advantageous research directions of China. Compared to plasmonics, phonon polaritons could be propagated longer in a more diffractionless fashion on chip, due to lower loss and its hyperbolic dispersion. Our work on bilayer MoO_3_ has pushed twistronics to twisted photons, and has been selected as Physics World Top 10 Breakthroughs in 2020.

For the first time, we have been able to apply the concept of moiré physics and of twistronics to the field of photonics and polaritonics, opening unique opportunities for extreme photonic dispersion engineering and robust control of polaritons on 2D materials. In order to unveil this paradigm, we used the discovered hyperbolic properties of MoO_3_ combined with topological photonic concepts. In particular, we demonstrate that, by pairing two thin MoO_3_ layers in close proximity to each other, and rotating one with respect to the other, highly unusual phenomena emerge. We show polaritons with robust dispersion properties that are controlled by a topological invariant, represented by the number of anti-crossing points in reciprocal space at which the dispersion bands of the isolated layers meet. When this integer changes from two to four, a topological transition must occur, yielding flat bands, reminiscent of magic-angle flat Fermi surfaces in twisted graphene bilayers, and supporting canalization for sub-diffractive imaging. These topological transitions occur at magic rotation angles, inherently robust to disorder, and determined by the opening angle of the hyperbolic bands of the isolated MoO_3_ layers. All these phenomena are inherently rooted in moiré physics here extended to the field of photonics. Our work introduces the theoretical foundations of this discovery, and experimentally observe, using near-field nano-imaging, highly tunable topological polaritons, sub-diffractive low-loss field canalization with resolution below λ_0_/40, and topological transition magic angles for phonon polaritons. We believe that these findings are truly important in the context of twistronics and valleytronics, nano-imaging, energy transfer, and quantum nano-optics.

After this work was completed, we realized that there was still a large space for improvement, and the loss of MoO_3_ was still relatively high. Not only our work, but also many other excellent works, have been limited to a propagation distance of 2–3 microns. Later, we discovered the ghost hyperbolic polariton in calcite crystals in the infrared band, which is a hybrid system of propagating waves and evanescent waves. We take the properties of calcite itself to polish a surface so that it has a specific angle with the optical axis. For the first time, we focused on this degree of freedom between the optical axis and the interface of the material, and realized the ghost hyperbolic polaritons, which can propagate nearly 20 microns on chip without diffraction. Calcite is a historical crystal. It is said that the Vikings used calcite to navigate in their early voyages. However, no one has ever paid much attention to the phonon effect of calcite in the infrared. It is the first time for us to utilize its hyperbolic dispersion property, and accidentally break the current bottleneck by discovering ghost phonon polaritons. Yet it is still a very challenging problem to freely structure polaritons and propagate in a curved path for instance.

**Q6: You published a review paper “Interface nano-optics with van der Waals polaritons” in** ***Nature*** **in 2021, which first proposed the concept of “interface polariton optics” and comprehensively described the latest progress in using interface optics to control van der Waals polaritons**^11^**. Could you talk about the current status and future development of van der Waals polariton control?**

**A6:** In 2021, we were invited by *Nature* to write a review paper. Some concepts or conjectures were proposed for the first time, such as how to control and manipulate the van der Waals polariton? New directions for interface nano-optics fusing meta-optics and van der Waals materials are proposed. For example, we can make the polaritons bend around to avoid some specific areas during the propagation process. It is found that many articles have gradually followed our call and realized with technical solutions.

**Figure:**
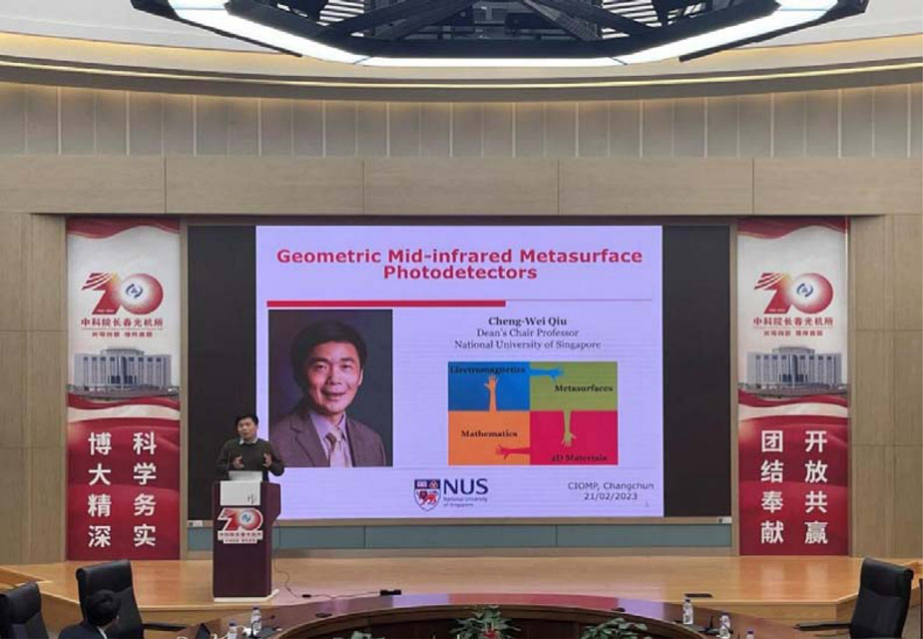
Prof. Cheng-Wei Qiu gave a scientific seminar in CIOMP

**Q7: In recent years, vortex light, as a special beam with helical phase potential wavefront and central phase singularity, has played an important role in advanced applications such as quantum optical communication, super resolution imaging, micro-nano particle control, and multi-channel information storage. At present, what are the commonly used methods to generate vortex beams? Your research work**^12^ **has achieved multi-channel OAM manipulation in both free space and near-field environments. Can you talk about the main advantages of this approach?**

**A7:** OAM is actually a fine physical dimension for channel multiplexing. Each topological charge is orthogonal, so the multiplexing has no limit in principle. The traditional method of generating vortex beams is usually to use the spatial light modulator, spiral phase plate, or metasurface. However, the spatial light modulator is very limited to pixel size. The spiral phase plate is thick and usually produces only one order. The vortex beam generated by the metasurface can form 2π or 4π phase variation by arranging the micro-nano structure. But how to generate multi-channel OAM simultaneously? Previous researchers achieved this by interspersing, that is, they use one array to form a specific order, and then insert another array through the gap, so it’s an assembly process in fact. Of course, the assembly inevitably leads to the decrease of efficiency. It is also impossible to assemble too densely, which would destroy the original single structural requirements. In this *eLight* paper, we realized multi-channel metasurfaces generating multiple OAM simultaneously in free space, without resorting to previous approaches. The original idea of this work came from the childhood experience when reading some science magazines. There were sections asking children to discern as many patterns as they could in a printed picture. Just like when we look at a sunflower, sometimes we can see different patterns. We have a bold guess, if light has a human-like vision, does it also have a visual sensation of overlapping multiple images? Hence we arrange nanoholes and exploit proximity effect achieved between adjacent nanoholes from two neighboring spiral trajectories. Experiments show this scheme indeed works well, and can be used for generating vortex beams in optics, which is a very novel way of generating multi-channel OAM in a limited area.

**Q8: As a star scientist in the emerging field of optics, can you briefly introduce your current research focus? What is the development plan for the future?**

**A8:** My current research focuses on structuring van der Waals layered materials and novel two-dimensional (2D) materials, integrating the concepts of symmetry breaking, discontinuous folding, and BIC of photonic crystals into 2D material systems. One paradigm is to directly combine the photonic crystal structure with 2D materials, and the other is to pattern 2D materials or layered materials directly into certain structures, which can synergize the advantages of 2D materials or layered materials in terms of nonlinear or photon emission, and the powerfulness in structuring at the same time. My future plan is to further develop such photonic van der Waals crystal concept in the optoelectronic detection at basic level and in the design of miniaturized light sources.

**Q9: You are not only an outstanding scientific researcher, but also have cultivated a large number of excellent students. Would you like to share with us your valuable experience in talent cultivation?**

**A9:** I find it difficult to summarize in this regard. Each student or young postdoc is unique, and there is no universal equation to apply. I need to understand their personalities, strengths, and weaknesses, so that I can give them some corresponding guidance. For example, for some very energetic and strongly-motivated students, I may suggest them to focus on short-range goals first. For some relatively introverted students, I may care them more and then gradually push their scientific research progress. I think there is one thing I can share: I don’t usually put forward quantitative metrics. I want to form a group that has reasonably healthy “competition” but also embraces the culture of mutual support and promotion of each other. This is a very personalized way of management. It is so lucky that most of the students or young researchers in my group are very excellent, basically, I don’t have to worry too much about these things.

**Figure:**
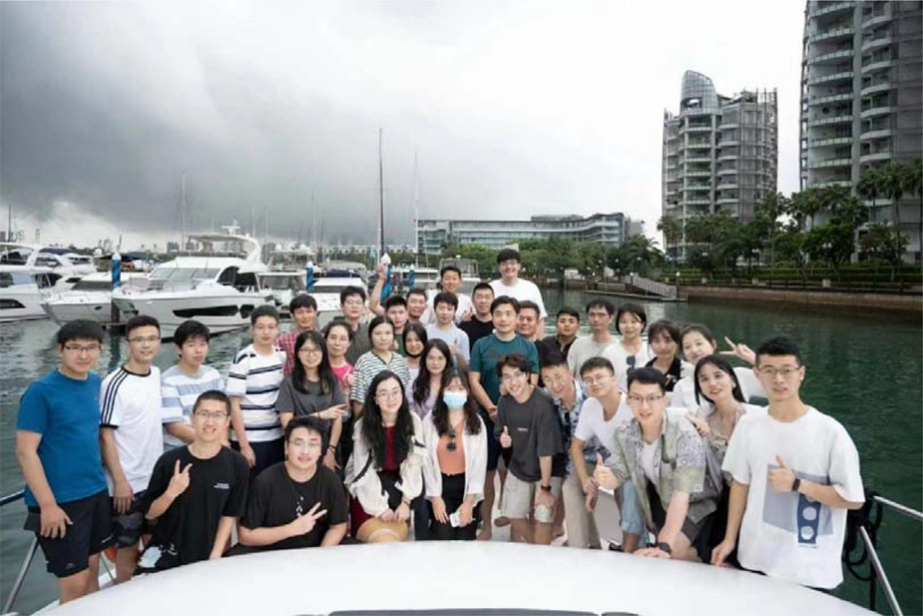
Prof. Cheng-Wei Qiu’s team celebrated the New Year

**Q10: You used to humorously call yourself “a light rider across disciplines”, which is highly consistent with the development orientation of** ***eLight*. As the co-Editor-in-Chief of this Excellent Action Plan High-starting Point Journal, what are the differences between** ***eLight*** **and other peer journals? What are your suggestions for** ***eLight*** **future development?**

**A10:** Ever since we started to create *eLight*, we have been asking ourselves a question: why we should have *eLight*? We find that the existing journals may be mainly categorized by disciplines and directions. But now optics has been incorporated in various fields, and we are excited to know how to promote and manifest the role of optics in different fields, and how to drive some new directions. For example, metamaterials have evolved from the initial optical field to mechanical, electronic, acoustic, and other fields. Another example is 2D materials, which was originally innovative in materials, but now it is also promising in optics. Therefore, *eLight* aims to explore the emerging directions of cutting-edge frontiers, hoping to bring a revolutionary influence to optics, as well as the cross fields of optics and other disciplines. In other words, we hope *eLight* can shine light into different fields. The current emerging sciences are largely produced in the integrated process across disciplines, so it is necessary to have some corresponding journals to serve these audiences and walk in the forefront of the existing cross disciplinary.

**Figure:**
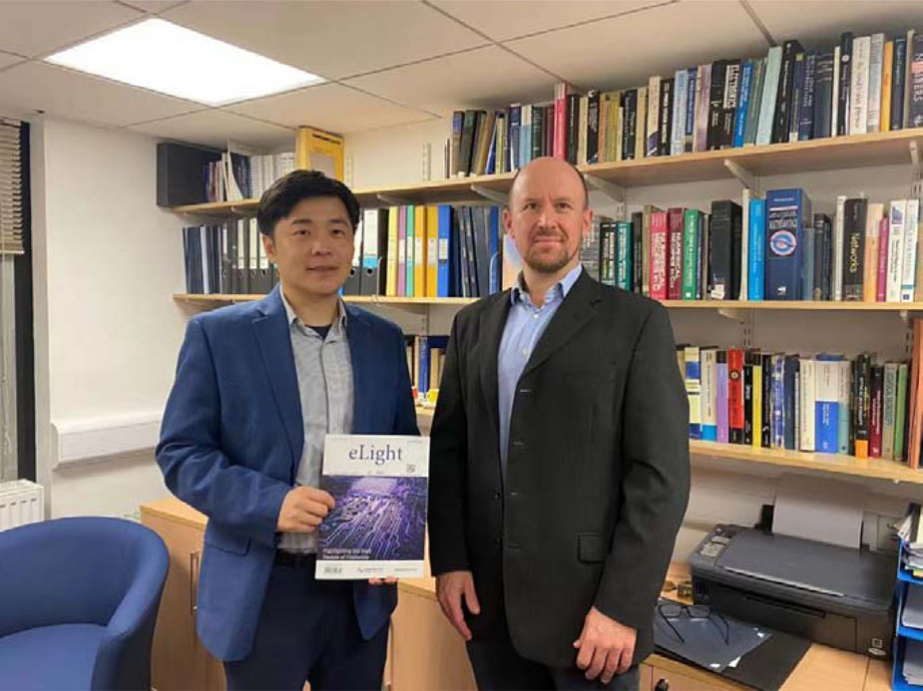
Prof. Cheng-Wei Qiu promoted *eLight*

**Q11: You have published many excellent papers in the world top journals including** ***Nature***, ***Science***, **and their sub-journals, and have been selected as Highly Cited Researcher by Web of Science for 4 consecutive years. How do you think of the relationship between scientific research and published papers?**

**A11:** I don’t think there is a “strong coupling” relationship between research work and published papers. It depends on the nature of your research work, basic, applied, or translational. For myself, if I do some basic and cutting-edge work, I will hope to share or report my findings through publishing papers. However, I think we should make a rational judgment on which journal we shall consider. First of all, you need to have an objective understanding of your work. If it is cutting-edge, you certainly want to have a larger audience to pay attention and follow-up. The basic logic is that your audience and related fields define where the paper is published. Nevertheless, I truly believe that if your works are sufficiently innovative and steadily maintain high quality, your paper will be seen wherever it is published. Don’t worry too much about this process.

**Figure:**
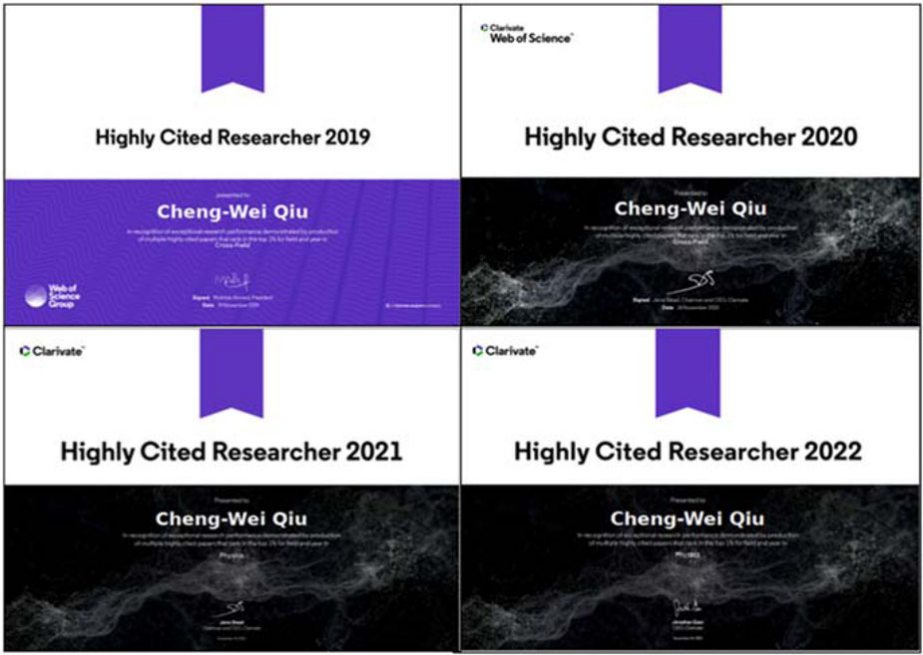
Prof. Cheng-Wei Qiu has been selected as Highly Cited Researcher for 4 consecutive years

**Q12: As we know, the scientific researchers are very busy with work. How do you balance work and life?**

**A12:** Now, the scientific researchers are indeed swamped with their work. In the mean time, they also have to take care of their families. Especially in some highly competitive scientific research fields, the pressure is intensive, because others may have achieved what you plan to do in a flash. In this case, how to balance work and life is very important. I think the core principle is: you have to make yourself happy. First of all, you should actively solve the difficulties and depression. Nevertheless, you can’t fully devote to work until having your family and life in order. When you are in a bad mood because of family or personal life problems, I think you are unlikely to be involved in scientific research effectively. In leisure times, you might engage in some entertainment activities including variety shows, movies, sports, or some social activities, etc., to make your mind unloaded for a while. Of course, there are different opinions on this balance, and everyone has his/her own way. For me, I sometimes watch some variety shows and TV dramas, including the recently popular TV series “The Knockout”, slow jogging, and talk shows. After those activities, I feel I am placed in a more comfortable state, and then become fully charged to engage with some difficult problems. I hope everyone could find their own way to achieve the balance between work and life.
